# An SDN-Based Solution for Horizontal Auto-Scaling and Load Balancing of Transparent VNF Clusters

**DOI:** 10.3390/s21248283

**Published:** 2021-12-11

**Authors:** Alejandro Llorens-Carrodeguas, Irian Leyva-Pupo, Cristina Cervelló-Pastor, Luis Piñeiro, Shuaib Siddiqui

**Affiliations:** 1 Department of Network Engineering, Universitat Politècnica de Catalunya (UPC), 08860 Castelldefels, Spain; 2 Bequant, 28003 Madrid, Spain; lpineiro@bequant.com; 3 i2CAT Foundation, 08034 Barcelona, Spain; shuaib.siddiqui@i2cat.net

**Keywords:** auto-scaling, bidirectional flow affinity, load balancing, NFV, SDN, transparent VNF

## Abstract

This paper studies the problem of the dynamic scaling and load balancing of transparent virtualized network functions (VNFs). It analyzes different particularities of this problem, such as loop avoidance when performing scaling-out actions, and bidirectional flow affinity. To address this problem, a software-defined networking (SDN)-based solution is implemented consisting of two SDN controllers and two OpenFlow switches (OFSs). In this approach, the SDN controllers run the solution logic (i.e., monitoring, scaling, and load-balancing modules). According to the SDN controllers instructions, the OFSs are responsible for redirecting traffic to and from the VNF clusters (i.e., load-balancing strategy). Several experiments were conducted to validate the feasibility of this proposed solution on a real testbed. Through connectivity tests, not only could end-to-end (E2E) traffic be successfully achieved through the VNF cluster, but the bidirectional flow affinity strategy was also found to perform well because it could simultaneously create flow rules in both switches. Moreover, the selected CPU-based load-balancing method guaranteed an average imbalance below 10% while ensuring that new incoming traffic was redirected to the least loaded instance without requiring packet modification. Additionally, the designed monitoring function was able to detect failures in the set of active members in near real-time and active new instances in less than a minute. Likewise, the proposed auto-scaling module had a quick response to traffic changes. Our solution showed that the use of SDN controllers along with OFS provides great flexibility to implement different load-balancing, scaling, and monitoring strategies.

## 1. Introduction

Technologies such as network function virtualization (NFV) [1] and multi-access edge computing (MEC) play a key role in the realization of 5G and beyond networks. NFV decouples the network functions’ (NFs) logic from proprietary hardware and runs them as software applications on general-purpose hardware [2]. This technology is expected to not only reduce capital expenditure (CAPEX) and operational expenditure (OPEX), but also improve business agility by introducing new ways to design, orchestrate, deploy, and manage network services (NSs). Additionally, MEC [3] brings computing, storage, and networking resources closer to the users.

The placement of applications and NFs, such as user plane functions (UPFs), at the network edge, provides significant improvements in the end-to-end (E2E) response time and bandwidth consumption, thus reducing the occurrence of bottlenecks in the network backhaul [4]. However, the application of optimized placement strategies along with the use of acceleration solutions is required to meet stringent 5G requirements for ultra-high bandwidth and ultra-low latency (e.g., high throughput with 1–10 Gbps and less than 1 ms response time in the data plane). A common approach to boost UPF performance is to integrate the UPF software networking elements with hardware-assisted network interface controllers (NICs), such as field-programmable gate arrays (FPGAs) [5]. An example of this is the solution design presented in [5], where UPF performance achieved more than 200 Gbps throughput with an average latency of 70 microseconds. This approach nevertheless restricts the number of available candidates for the deployment of UPFs due to their dependency on hardware capabilities (e.g., availability of FPGA-NICs). To overcome this limitation, edge applications and NFs can make use of hardware capabilities or virtualized accelerators available in remote locations (e.g., the cloud). However, the latter may produce network overheads, which may affect bandwidth-bounded applications [6]. In this context, the combination of UPFs and virtualized traffic accelerator capabilities at the edge [7] could help to reduce bandwidth congestion, packet loss and latency.

Despite this potential solution, the deployment of virtual network functions (VNFs) at the edge poses several challenges due to the fact that they are more sensitive to traffic variations and the limited resources of the MEC nodes [8]. When the allocated resources are insufficient to meet the requested demands, the user’s perceived quality of service (QoS) degrades [9]. In contrast, if the assigned resources are over-provisioned, the service-associated costs increase. To make efficient use of MEC resources while providing the high QoS that characterizes 5G networks and lowering the service provisioning cost, the dynamic placement readjustment of VNFs along with the scaling of their assigned resources (e.g., number of instances or capacity) based on their workloads or user’s traffic demands is imperative.

The virtualization of some traditional NFs such as traffic accelerators or firewalls, which can be deployed in a bump-in-the-wire (BITW) manner to avoid altering the communication endpoints, would bring about extra difficulties. No modification in the packet headers is allowed when redirecting traffic to guarantee the standard functionality of a service (e.g., avoid service interruption) due to their transparent deployment. Scaling out BITW VNFs may cause occasional loops in the network and subsequent problems (e.g., slow and irregular connections and system failures). Additionally, bidirectional flow affinity during the flows’ lifetime may also be required.

Most of the literature addressing the problem of load balancing (LB) and auto-scaling of VNF clusters [10,11] neglects the aforementioned use case scenario and its particularities. To the best of our knowledge, we are the first to tackle the problem of dynamic auto-scaling and load balancing of transparent VNFs while ensuring bidirectional flow affinity. In this regard, the key contributions of this paper can be summarized as follows:

1. The design, implementation, and evaluation of a solution to make load balancing and auto-scaling decisions to manage a cluster of transparent VNFs.
2. The proposed solution can not only distribute traffic according to the selected LB strategy but also guarantee bidirectional flow affinity without packet modification.
3. An auto-scaling strategy is presented to manage the size of a transparent VNF cluster.
4. The testbed of the proposed strategy in this research utilized real hardware unlike most works on this topic, which use simulation tools (e.g., Mininet) to assess the performances of their proposed strategies.

The remainder of this paper is organized as follows. Section 2 introduces some works related to the load balancing and auto-scaling of VNFs. Section 3 gives some background on technologies, such as Open Source MANO and software-defined networking (SDN). In Section 4, a solution is proposed for traffic distribution and the auto-scaling of a cluster of transparent VNFs is presented, and Section 5 validates its effectiveness. Finally, Section 6 concludes our work and presents future work directions.

## 2. Related Work

This section provides a review of the literature related to load-balancing and auto-scaling solutions in virtual environments.

### 2.1. SDN-Based Load Balancers

Most SDN-based load-balancing studies focus their solutions on the designing of new methods to decide on the most suitable destination for a given traffic flow. In this manner, the authors of [12,13] propose SDN load-balancing algorithms to distribute requests to servers dynamically. Their proposed strategies select the most suitable server based on the real-time metrics of CPU utilization, memory utilization, and disk utilization. In [12], the least loaded server is selected to handle traffic requests, whereas in [13] a dynamic weighted random selection algorithm (DWRS) is used. The DWRS gives higher weights to underutilized services, so the server with the least load has a higher probability of being chosen. Chen et al. [14] propose an SDN-based load-balancing solution to distribute traffic among a server cluster. Clients’ requests are sent to a virtual IP address and the traffic is then redirected to the selected server. Like traditional load balancers, Chen et al.’s solution implies using a virtual IP and modifications of packets for routing the traffic to the servers.

In [15], two OpenFlow-based mechanisms are presented for balancing multi-path TCP (MPTCP) traffic to a pool of servers. Though the proposed solutions guarantee the selection of the same server for subflows belonging to the same MPTCP session, it is at the expense of packet modification. Similarly, the solution of Ma et al. [10] relies on OpenFlow switches (OFSs) to reroute traffic dynamically inside the network function chaining (NFC). The NFC management uses a unique pair of VLAN tags for each chain. By using the open-source PF_RING network socket [16], the proposed solution captures incoming packets and adds the VLAN tags. At any time, the control element can update the number of used VLAN tags for traffic balancing.

The authors of [17] propose a load-balancing mechanism to distribute traffic flows among switches by considering the equipment singularities. A local SDN controller guides a data plane scalability mechanism to monitor the network traffic and used resources on the switches. This mechanism adjusts the number of active switches to meet network demands. Moreover, the load balancer configures the required flow rules on target switches and relocates flow rules to improve resource usage.

Abdelltif et al. [18] propose an SDN-based load-balancing service to optimize resource utilization while reducing the user response time. The proposed mechanism comprises three application modules (i.e., service classification, dynamic load balancing, and monitoring) that run on top of an SDN controller. The dynamic load balancing module distributes the incoming traffic to the servers according to the service type. The feasibility of the proposed scheme is proved through experimental findings.

### 2.2. Load Balancing and Auto-Scaling

The authors of [19] provide insights into the network service descriptors (NSD) by addressing fields related to scaling actions. Hinojosa et al. highlight the importance of a proper NSD configuration to ensure the appropriate scaling operations of VNFs. Additionally, they offer an overview of the different available scaling procedures in NFV and how to trigger them in an automated manner.

In [20], an auto-scaling approach for 5G data plane (DP) VNFs based on throughput utilization is presented. This approach makes scale-out decisions based on the utilization threshold, while scale-in events are run periodically to remove empty DP functions. Incoming user requests are assigned to the DP with the highest throughput. Alawe et al. [11] present an algorithm based on control theory for the horizontal scaling and load balancing of 5G Access and Mobility Management Function (AMF). Additionally, scaling actions are triggered according to the traffic load.

The authors of [21] propose a threshold-based scaling mechanism that considers workload variations of the evolved packet core (EPC) to perform scaling actions. Its proposal is formed by three modules: data collection, scaling decision, and scaling execution. To evaluate their horizontal scaling procedure, the authors deploy a set of clusters for different EPC elements. Each cluster contains a load balancer that uses a round-robin algorithm to distribute the traffic among the cluster’s instances.

Dutta et al. [9] present a solution for the dynamic and automatic scaling of VNFs. Their main aim is to ensure efficient resource utilization while improving the quality of experience (QoE) of the offered services. In their solution, the incoming traffic is distributed to the pool members through an LB with a round-robin configuration. However, this approach implies packet modification since the LB works as a front-end service that reroutes incoming requests. Ma et al. [10] propose a generic solution for the horizontal scaling of VNFs. Their solution relies on two load balancers (a master and a salve) to distribute the traffic in the uplink and downlink directions. Moreover, they also address the flow affinity problem when balancing traffic among VNFs. To solve the problem, they use connection-aware traffic load balancers based on a hashing function to maintain affinity between connections and NFs. The authors of [22] demonstrate the importance of jointly considering instances of load balancing and auto-scaling of VNF. They highlight the necessity of designing these policies so that they are aware of one another to improve QoS and ensure a more efficient use of resources.

Lange et al. [23] proposes an architecture for VNF that exploits the benefits of network softwarization and machine learning-based approaches to orchestrate and manage the life-cycle of VNFs. By monitoring the deployed VNF’s state and predicting its resources, the proposed solution can perform scaling actions in advance while load-balancing actions are leveraged to OpenStack load-balancing features. However, the auto-scaling of VNFs and load-balancing performance are not covered in the obtained results. Thus, further experiments are necessary in order to evaluate the overall feasibility of their proposal.

Despite the proposed solutions, none of the aforementioned works address the issue of load balancing and auto-scaling transparent VNF clusters. Of these works, the most similar to this paper is [10], as it presents a solution to address both issues by taking into account bidirectional flow affinity. However, its solution implies packet modification, which is not convenient for scenarios with BITW deployments. This type of deployment imposes additional challenges in terms of scalability.

## 3. Background

This section presents some background on NFV management and orchestrators, SDN, and OpenFlow switches. These elements are fundamental pillars for our solution implementation.

### 3.1. Open Source MANO

NFV is considered a key enabler technology for addressing the stringent requirements of 5G and beyond networks [24,25,26]. Its agility and flexibility to manage network resources and services support carriers in deploying a variety of verticals with different requirements while reducing costs. In this vein, the ETSI NFV Management and Orchestration (MANO) framework [27] provides a standard architecture, used as a reference by vendors and open source MANO projects for the monitoring and provisioning of VNFs [28]. The group of open source MANO includes several projects such as Open Network Automation Platform (ONAP) [29], Open Source MANO (OSM) [30], Open Baton [31], and Cloudify [32]. Of these projects, ONAP and OSM are the most prominent ones in both academic and industry sectors since big operators such as AT&T and Telefonica support their development [33].

This paper adopts OSM as the NFV orchestrator (NFVO) and virtual network function manager (VNFM). It is responsible for the deployment of NSs and VNFs and monitoring the life-cycle management of VNFs. The selection of OSM was based on its maturity, performance, and comprehensive utilization [33]. Another criterion for this selection was its modular architecture formed by several elements, including Resource Orchestrator (RO), Lifecycle Management (LCM), Policy Manager (POL), and Monitoring (MON). Thus, our implementation could obtain information related to NSs and their associated metrics by directly communicating with the modules in charge of managing this information (i.e., RO and MON modules).

### 3.2. Software-Defined Networking

Along with the NFV paradigm, SDN is recognized as a crucial pillar in the development of 5G and beyond networks to fulfill their network requirements [24,25,26]. The SDN architecture is composed of three layers: application, control, and data [34]. The control plane is formed by the controller, which manages all the devices (e.g., routers and switches) in the data plane in a unified manner. Additionally, network applications, used by the controller, are implemented and executed in the application layer. One of SDN’s main features is the control and user plane separation (CUPS). The CUPS guarantees that the resources of each plane can be scaled independently. It also allows the placement of the user plane functions closer to users, thereby reducing network response times and bandwidth consumption. Moreover, the separation of these two planes allows the direct programmability of network policies, thus ensuring simplicity and versatility in networking configurations [34]. Additionally, SDN offers new methods to flexibly instantiate NFs and services while reducing expenses and boosting performance. For instance, it allows the support for new protocols and the ability to adapt network resources and topology to changes in the configuration and placement of NFs and services [35,36].

The SDN concept is applied in a wide variety of solutions in which the controller is a key element in the control operations. In this regard, the architectural deployment of the controllers is crucial to guarantee that the performance of the overall network is adequate. Specifically, from an architectural point of view, SDN controllers can be classified into two groups: centralized and distributed. The centralized architecture is formed by a unique controller for the sake of simplicity. However, this design represents a bottleneck in the network and has scalability limitations when the network traffic increases. By contrast, a distributed architecture improves network scalability, flexibility, and reliability. For instance, this architecture avoids having a single point of failure since when one of its distributed controllers fails, another(s) can assume its functions and devices. Therefore, this design is more resilient to different kinds of disruptions. Furthermore, with the programmability of SDN, this process can be automated and configured according to the design and requirements of the network. Thus, the distributed architecture is capable of responding and adapting to new requirements and conditions.

### 3.3. OpenFlow Switches

An OFS consists of one or more flow tables and a group table, which perform packet lookup and forwarding, and one or more OpenFlow channels to communicate with an external controller. The switch communicates with the controller, and the controller manages the switch via the OpenFlow protocol [37]. The controller can add, update, and delete flow entries from the switch flow tables through the OF protocol. A flow entry is formed by several fields such as match, counters, actions, and priority. Specifically, the match field allows the creation of flow entries according to various match criteria. For instance, it may be configured to match various packet header fields (e.g., source/destination IP/MAC/ports, protocol, VLAN ID), packet ingress port, or metadata value.

When a flow arrives at an OFS it verifies, in its flow tables, if there is already a defined rule that matches the incoming flow. If such a rule exists, then the specified actions are executed. A wide variety of instructions can be applied to flow entries, such as packet forwarding (e.g., forward packet to a port) and packet modification. In the case of several rules matching the flow description, the rule with the highest priority order is applied. In contrast, if no match is found in any flow tables, the switch can be configured to send the packet to its assigned controller or drop it.

### 3.4. Flow Affinity

Utilizing flow affinity implies that packets belonging to the same flow will undergo similar treatments along their paths (e.g., processed by the same set of network functions) [38,39]. In this study, we define bidirectional flow affinity as the property of processing packets associated with a specific flow by the same network function, in both directions of their data path (i.e., uplink and downlink). Traffic flows are identified by five parameters from the packet headers: source IP, destination IP, protocol, source port, and destination port. These parameters form a five-tuple, and by combining them, different hashing methods can be defined. The three most popular combinations are: source-destination IP, source-destination IP + protocol, and source-destination IP + source-destination port + protocol.

## 4. Solution Proposal

In this section, after a brief overview of the problem of interest, the proposed solution for the management (i.e., balancing and horizontal auto-scaling) of a cluster of transparent VNFs is presented.

### 4.1. Problem Description

Network operators are virtualizing their physical network functions to align with new services requirements imposed by 5G networks and beyond. These NFs can be grouped into different categories according to their characteristics (i.e., by network functionalities and connectivity, network security, and network performance). Many of the NFs associated with network performance (e.g., traffic shaping, rate limiting, and traffic accelerators) are usually transparently placed between the access and internet network providers. In other words, they do not have a forwarding IP since they are placed as BITW functions and, thus, work in layer 2 of the OSI model. The virtualization of NFs offers flexibility for the management of network resources since NFs can be scaled as their processed traffic changes. Existing MANO frameworks (e.g., OSM) offer some scaling policies to automatically adjust VNF capacity to traffic demands. However, the scale-out policy of MANO frameworks is characterized by the placement of new deployed functions in the same subnetwork as the original ones. Moreover, they do not allow for the modification of previously launched NSs, for instance, the adding of new VNF instances or subnetworks. These limitations cause severe problems in the network when scaling transparent functions since network loops may appear.

Load balancers are crucial to avoid overload and guarantee efficient traffic distribution to the pool members when more than one instance of a given type is deployed and forms a cluster. Additionally, load balancers enhance the availability and reliability of the network service by redirecting incoming requests to only healthy VNFs. Well-known MANO frameworks, such as OSM and ONAP, lack native load-balancing services. Thereby, they rely on specific virtual functions (e.g., HAProxy [40]) that need to be deployed along with the pool members to provide this feature. However, this solution requires the assignment of a virtual IP to the load balancer to redirect all the incoming traffic to it. The latter is recommended when the pool entities are a final service or the destination of the incoming traffic. Otherwise, an extra function may need to be inserted in the flow path to modify the header packets to reach their final destination. A similar shortcoming has been found in virtual infrastructure management (VIM) technologies, such as OpenStack. Though these technologies provide load-balancing services (i.e., Octavia which is based on a neutron load-balancing mechanism (load balance as a service, LBaaS)), they are also aimed at final services.

Moreover, some VNF types, such as firewalls and intrusion detection systems, require that the same instance process all the fragments of a given flow during the flow lifetime. Other VNF types (e.g., TCP traffic accelerators) may need to ensure bidirectional flow affinity to work properly.

Under these circumstances, designing a mechanism for the efficient management of transparent VNF clusters is critical. The envisioned solution must be able to dynamically scale cluster resources, balance the flow traffic between transparent VNFs, ensure bidirectional flow affinity, and avoid packet modifications and extra processing.

### 4.2. Design Architecture and Implementation

**Figure 1:**
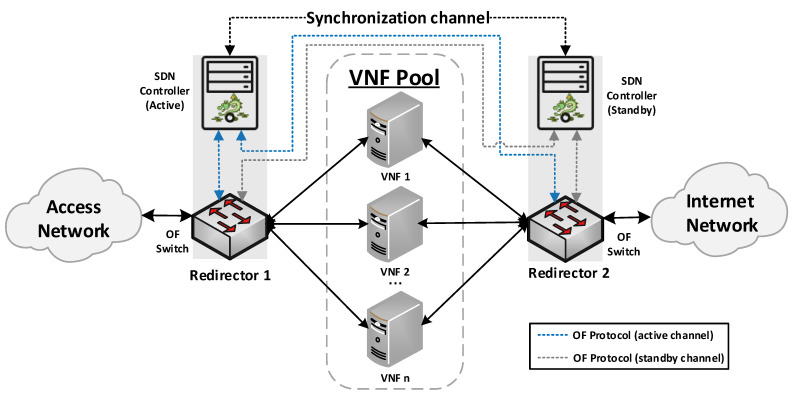
A high-level view of the proposed solution.

Our proposed solution for the management of transparent clusters is formed by two redirector VNFs, one at each side of the cluster. The reason for placing redirector functions on both sides of the cluster (uplink and downlink directions) is to ensure the bidirectional flow affinity requirement (see Section 4.2.3). Each VNF redirector consists of an OFS and an SDN controller mainly responsible for load-balancing aspects. The SDN controllers work in an active-standby configuration. The active controller replicates the network information to the standby one by using the data distribution service (DDS) method described in [41]. Since the DDS method is based on the publish/subscribe paradigm, the active controller will publish the discovered nodes and configured flows through a data stream also known as a topic. Specifically, the used topic is composed of seven fields: Identifier, NodeId, Port, SourceNode, SourceNodePort, DestinationNode, and DestinationNodePort. Using the identifier field, the active controller announces whether it will send a node or a flow. In the case of the standby controller, it will be subscribed to the used topic, thus receiving the network information. This approach avoids the network discovery phase of the standby controller in the case of a failure in the active controller, thus reducing the service downtime and improving the fault tolerance of the system. The master controller is placed facing the access network to reduce response time, since most traffic requests originate in the uplink direction. The use of two SDN controllers is not strictly necessary, but it is highly recommended to improve the robustness of the solution. Figure 1 provides a general overview of the proposed design as well as the interconnection mode among the solution elements.

**Figure 2:**
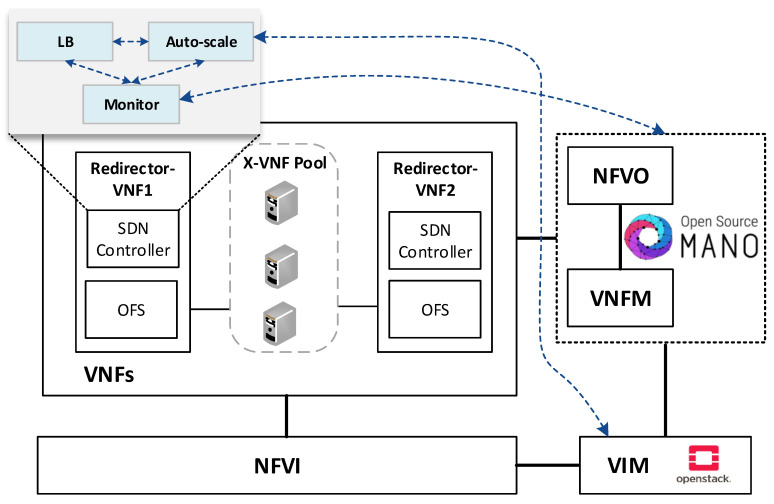
An overview of the solution components and its communication with the NFV entities.

The envisioned VNF redirector application has a modular design, where each module performs a specific function (i.e., monitoring, auto-scaling, and load balancing). The logic of these functions runs on top of the SDN controllers, as shown in Figure 2. The monitoring module is in charge of checking the status of VNF instances as well as periodically collecting metrics. This information is used as input by the load balancing and auto-scaling blocks to perform their logic. The auto-scaling module is in charge of managing the cluster size. Specifically, it triggers scale-in and scale-out actions based on the status and load of the pool members. Furthermore, the load-balancing module is the core of the redirector application. It is responsible for selecting the VNF instance to which a traffic request will be assigned for its processing. The selection of a VNF instance depends on the specified load-balancing strategy (e.g., least loaded CPU) and the status and capacity of the cluster’s members. Each instance belonging to the VNF cluster is associated with a unique port in the OF switches. To simplify the solution complexity, a mirror configuration, in which the same port in both switches is selected, is recommended.

To improve the VNF redirector performance and allow faster packet processing in the load-balancing module, our design exploits the parallel processing capacity of multi-cores along with a multi-threaded technique, thus avoiding delay in its procedure due to the performance of other modules. The following subsections provide more insights into the operation mode of the VNF redirector’s modules.

#### 4.2.1. Monitoring Module

Transparent VNFs are not compatible with well-known health monitor methods, such as PING, TLS-HELLO, UDP-CONNECT, and TCP-CONNECT, since they all require that their queries be directed to an IP address. A workaround could be the use of the management network to send the messages associated with one of these health methods. However, this implies additional load in the VNF cluster. To resolve this shortcoming, our solution uses information already available to other components (i.e., VNFM). The VNFM monitors the health status and performance (e.g., CPU and memory utilization) of VNF instances and network services to manage their life cycles. In the OSM MANO, its monitoring module has a feature (mon-collector) to collect the specified metrics in the VNF descriptors. The mon-collector polls the VIM, where the VNFs are deployed, to gather the desired metrics and stores them in its Prometheus time-series database (TSDB). To make this information available to the redirector application, our solution implements the monitoring module. This module communicates with the Prometheus TSDB and gathers VNF status and metrics. In this manner, the LB can be aware of the healthy and unhealthy instances and their available resources. This approach solves the limitation mentioned above and avoids overloading the cluster with frequent health polling through the management network.

This monitor module also gathers information about the deployed NS configuration, for instance, the ID of the virtual deployment units (VDUs) that constitute the transparent cluster and their subnetworks. These data are used to match the VDUs with the OVS switch interfaces to which they are connected. These interfaces are discovered upon the OFS registration on the SDN controllers. In this way, the monitor module helps the redirector application to discover the network topology.

Additionally, when the monitoring module detects an unhealthy instance, it communicates the LB to trigger a flow rule updating process. Then, the LB module uses a method to delete the OF rules associated with the unhealthy instance in the switches table. This is achieved by sending a delete flow message to the OF switches, which instructs them to delete the flow rules that contain the specified port (i.e, the one connected with the unhealthy instance). With this action, the monitoring module avoids network disruption because another healthy transparent VNF can be selected by the LB to process the incoming traffic.

#### 4.2.2. Auto-Scaling Module

The automatic scaling of transparent VNFs imposes several challenges to loop avoidance. For instance, when more than one transparent VNF is deployed in the same subnet, loops appear given that they act as a "wire" in the network. Thus, transparent VNFs should be deployed on different subnets to avoid network loops and redirect flows to a specific transparent instance since they do not dispose of routing information. However, the current MANO frameworks do not allow us to specify different subnets when executing auto-scaling policies or to add new VNF instances and subnetworks to already instantiated NSs. Therefore, we diverge from the assumption that the VNF cluster has been already dimensioned to resolve these challenges. In other words, the cluster is launched with all its members, and each VNF instance connects with one interface in the OFS. It should be noted that the OFS interfaces also need to be dimensioned according to the pool size. However, to save energy and computing resources, only the minimum required number of instances remains active, and the rest are on standby. Thus, the main aim of the scaling module is not to create or remove instances, but to manage their status (i.e., activate or deactivate them). To this end, the auto-scaling module needs to interact with the VIM. The OpenStack VIM, for instance, through its Nova service [42], offers several options to manage VNFs such as stop or start, suspend or resume, shelve or unshelve, pause or unpause and resize. It should be further noted that each of these options has a different impact on the resource utilization of the system as well as the VNF activation time.

For the implementation of the auto-scaling logic, the following parameters need to be defined.

- Scaling metric: the metric to be monitored (e.g., CPU or memory) and upon which scaling actions will be taken;
- Aggregation type: refers to how the scaling metric is gathered (e.g., average or maximum values);
- |VNF|_max: maximum number of active instances in the cluster;
- |VNF|_min: minimum number of active instances in the cluster;
- Thresholds: upper and lower bounds of the selected metric upon which scale-out or scale-in actions are triggered, respectively;
- Threshold time: a minimum amount of time in seconds during which the state of the scaling metric with regard to the threshold values must sustain to trigger a scaling event. Different threshold times can be defined for scale-in and scale-out actions;
- Cooldown time: the minimum amount of time that the system must wait after triggering an event before activating another.

**Figure 3:**
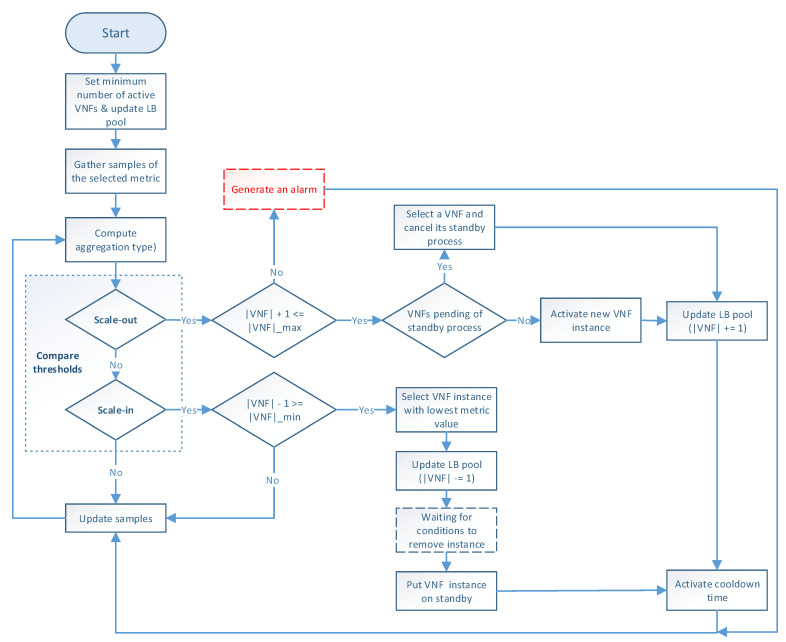
Flowchart of the proposed auto-scaling procedure.

The auto-scaling logic runs during the system lifetime and updates the scaling metric values at each time instance. Figure 3 illustrates the programming logic of the auto-scaling block. It starts by setting the minimum number of active members and exposing this updated information to the LB block. Next, it proceeds to collect samples of the specified metric until completing a threshold time, for which communication with the monitor block is required, and computing its aggregation type with regard to the set of active members in the VNF pool. Afterward, the auto-scaling conditions are verified by comparing the obtained value with the maximum and minimum thresholds. If one of these conditions is met, the effect of its respective scaling action on the number of active instances is verified before proceeding with the activation or deactivation of a VNF instance. The latter helps to maintain the number of active instances between the established values. If the scaling action implies the violation of any of these thresholds, the scaling procedure is discarded, and the system continues gathering samples. Additionally, when the scaling procedure is omitted because the maximum number of active instances has been reached, an alarm can be activated to notify the system administrator that further actions need to be taken. Otherwise, new instances can be started, or existent ones deactivated depending on the triggered condition. According to the VNF service type, a waiting time may be required before putting an active instance on standby to prevent service degradation. In the case of scale-out actions, existing VNFs waiting to change their status to standby are selected instead of activating new ones, thus canceling their associated standby process. After each scaling decision, the set of available VNF instances is reported to the LB. It must be noted that this set is immediately updated for scale-in actions to avoid the assignment of new flows to a VNF instance in a standby process. Finally, a cooldown timer is activated to prevent unnecessary scaling actions caused by possible system instability, and the set of gathered samples is updated.

#### 4.2.3. Load Balancing Module

There are two ways for maintaining flow affinity when load balancing traffic. One approach uses a dedicated load-balancing algorithm based on an IP hashing method. The other uses a stick table in memory along with a non-deterministic LB algorithm (e.g., round-robin or least connections). The use of the load-balancing algorithm approach is suitable as long as the number of involved instances does not change. Otherwise, more complex techniques, such as consistent hashing, need to be implemented to diminish the effects of variations in the number of instances on the flow affinity mapping. This method is recommended for load-balancing applications that do not require synchronization among LBs but still use the same hashing function. The second approach requires a global view of the system or synchronization among the LBs to ensure bidirectional affinity. The advantage of this approach is that no session is redirected when a new instance is added to the cluster. Our solution is based on the second technique since flow affinity is guaranteed upon flow entries registered in the OFS tables. Moreover, our solution does not require any exchange of information between the LBs (i.e., switch tables). The reason is that the flow entries associated with a given flow are simultaneously created in both switches by their master controller, which has a global view of the cluster. A drawback of working with flow tables is that flow waiting time may increase with significant traffic. Nevertheless, this effect can be diminished by implementing an efficient mechanism to manage switch tables [43,44].

Additionally, old flow entries belonging to expired flows can be removed to avoid overloaded flow tables. Using the idle_timeout field in OF FLOW_MOD messages is highly recommended in this process. This parameter allows you to specify a time interval that the switch can use to remove idle flow entries when no packet has matched within the given number of seconds. For this process to be completed successfully, the OFS must keep track of the arrival time of the last packet associated with the flow.

**Figure 4:**
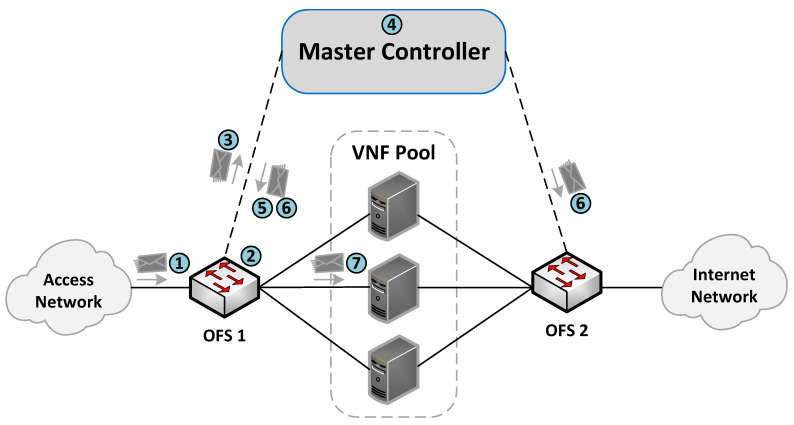
Communication process between the master controller and the OFSs.

The communication process between the OFSs and the master controller for the flow rules creation is described in Figure 4. More specifically, when an incoming flow arrives at an OFS (step 1), the switch searches in its flow tables the existence of a rule matching the flow (step 2). If there is a match, the switch applies the actions associated with the flow (step 7). Otherwise, the switch sends the packet to its assigned master controller through an OF PACKET_IN message (step 3). The controller is responsible for extracting the header packet information and determining the actions that need to be applied to the flow (step 4).

**Figure 5:**
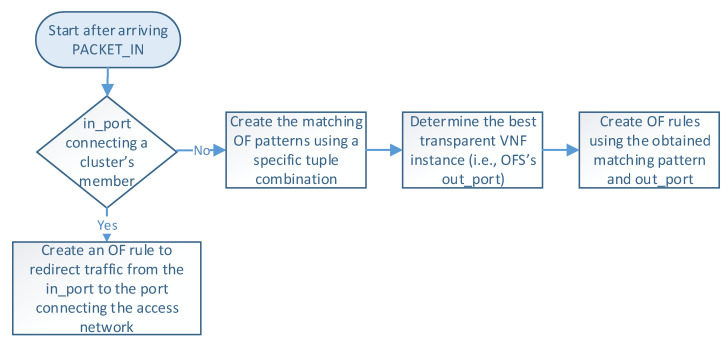
Flowchart of the proposed load-balancing procedure.

Figure 5 describes the logic executed in the SDN controller when it receives a PACKET_IN message (i.e., step 4 in Figure 4). The controller begins by reading the packet information (e.g., headers and OFS’s in_port), which determines how the flow is processed. At this step, there are two possible options according to the packet’s in_port in the OFS. More specifically, when OFS’s in_port connects with a cluster member, an OF rule is configured to redirect the flow traffic from the cluster member port to the one connecting the access provider network. This rule is created only once during the operation phase unless specified in the idle_timeout field. On the other hand, if the in_port is the one connecting the access provider network, the controller configures actions based on the packet’s header information. In the last case, the load-balancing module creates an OF matching pattern using a specific tuple combination (e.g., source IP, destination IP, protocol, source port, destination port). Several treatments can be defined according to the different tuple combinations and the values of their parameters. For instance, flows matching a given protocol type or source IP can be configured to be processed by a specific cluster member by default. In contrast, other flows may require the selection of an appropriate VNF instance for their processing. Afterward, this module determines the switch’s out_port by selecting the best VNF instance according to the specified load-balancing strategy. Then, the controller creates an OF rule with the selected matching pattern and the obtained out_port. Please note that each transparent VNF instance connects to a specific switch’s port.

Once the controller has determined the OF rule, it sends an OF PACKET_OUT message and a FLOW_MOD message to the switch that sent the OF PACKET_IN (steps 5 and 6). These steps instruct the switch to send the packet through the selected port and configure the specified rule in its table. Similarly, a reverse OF rule is proactively configured in the opposite switch by swapping the source and destination fields in the matching pattern and specifying the same action (i.e., OFS’s out_port). In this manner, our load-balancing module ensures bidirectional flow affinity by simultaneously creating flow entries in both switches.

Currently, the load-balancing module has available three load-balancing strategies: random, round-robin, and least loaded [14,45]. The random approach is the simplest one since the controller only needs to be aware of the active members in the pool. This information is used by the controller to randomly choose a VNF instance to redirect incoming flows. Round-robin is one of the most popular load-balancing algorithms due to its simplicity. It distributes incoming traffic requests by sequentially selecting an active pool member to process the incoming traffic. Thus, each time that a new PACKET_IN arrives at the main controller, a different member is selected. Once all the members have been analyzed, it starts rotating again by selecting the first active VNF in the list. Finally, the least loaded algorithm is based on the load utilization (e.g., CPU or memory) of the active VNF members. These data are obtained from the monitoring block and used to select the active instance with minimum load to process new flows.

## 5. Evaluation

The main objective of this section is to validate the proposed solution as a feasible approach to manage traffic distribution and resources assigned to a cluster of transparent VNFs according to dynamic traffic demands.

### 5.1. Experimental Setup

**Figure 6:**
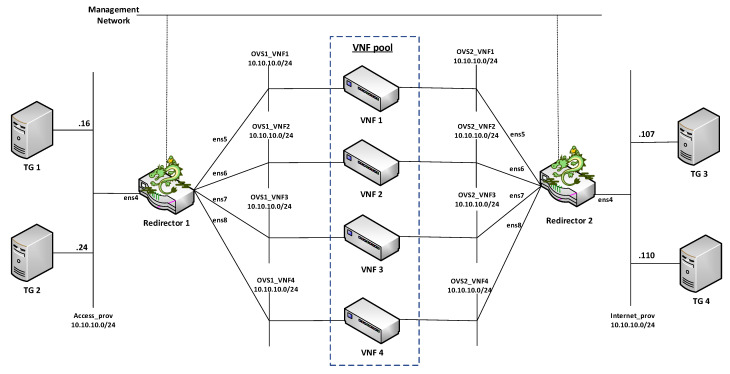
Simplified overview of the network service topology.

**Figure 7:**
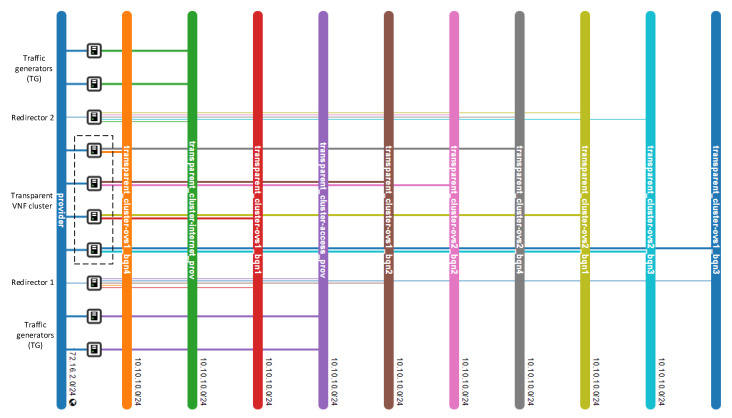
Network topology overview of the test scenario in OpenStack.

For the validation of the proposed solution, a simplified scenario was considered. The network service consisted of a VNF cluster, two redirector VNFs, four traffic generators (TGs), and one network. The cluster was formed by four generic VNFs that were transparently deployed by configuring their interfaces as a bridge. The TGs’ main function was to inject traffic between the access and data networks. This traffic had to traverse the cluster in a distributed manner. Additionally, subnetting was applied to divide the network domain into smaller subnets. This division allowed each group member to be deployed on a different subnet. It thus ensured that traffic passed only through a single transparent VNF and E2E connectivity between TGs located on different sides of the group. However, adopting the subnetting can introduce several loops in the network when working with BITW VNFS. To account for this, the network service deployment was automated to enable the spanning tree protocol (STP) in the redirectors and transparent VNFs interfaces to avoid network outages due to loops. Once the master controller had taken over the network control, the STP was deactivated to enable interfaces with blocked status. Figure 6 and Figure 7 illustrate the NS topology in a simplified overview and in an OpenStack view, respectively.

For our implementation, Ryu [46] was selected as the SDN controller due to its simple configuration and Python implementation that allows for its integration with the OSM client v.8.0.4. This client is used to gather information about VNF instances through the OSM framework. For the OFSs, we used Open vSwitch v.2.15.1. These switches communicated with the controller in the out-of-band mode because we used a separate network (i.e., the management network) to connect forwarding devices to the controller and exchange control traffic. Table 1 summarizes the hardware and software specifications (version and properties) of our testbed.

### 5.2. Solution Validation

**Figure 8:**
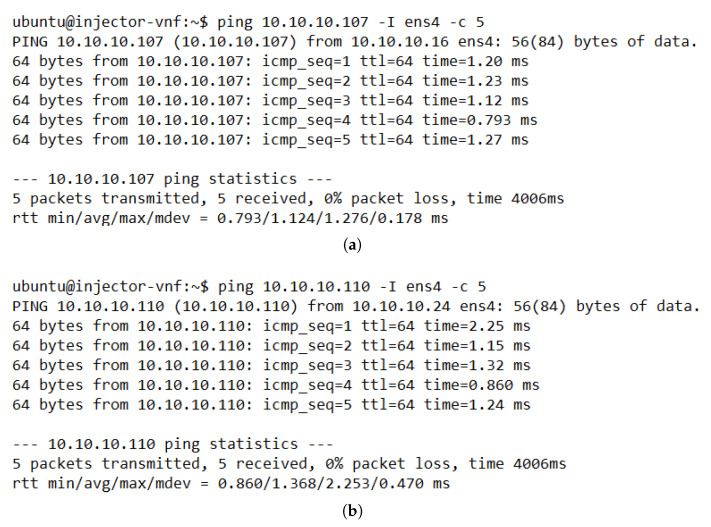
Command line output for ping tests between TGs located in different subnets. (**a**) Results of ping test with TG 1 (IP: 10.10.10.16) as source and TG 3 as destination (IP: 10.10.10.107). (**b**) Results of ping test with TG 2 (IP: 10.10.10.24) as source and TG 4 as destination (IP: 10.10.10.110).

As a proof of concept, we ran several experiments to verify the correct operation of each aspect of the proposed solution. We started by checking the connectivity between the different elements of the system. Figure 8 depicts the results of the ping tests between traffic generators located at different ends (access and internet sides) of the VNF pool. The successful ping execution showed stable E2E traffic through the cluster and low values of time response, which indicates the absence of loops in the network. Moreover, these results showcased that our solution was working as expected since the module related to the flow configuration in the OVSs (i.e., the load-balancing module) configured the required rules to guarantee E2E traffic without losing any packet. Additionally, the ping test also evidenced that the monitoring module had updated information about the active cluster members, as the load-balancing module selected a healthy instance to configure the flows in the switches.

#### 5.2.1. Bidirectional Flow-Affinity

**Figure 9:**
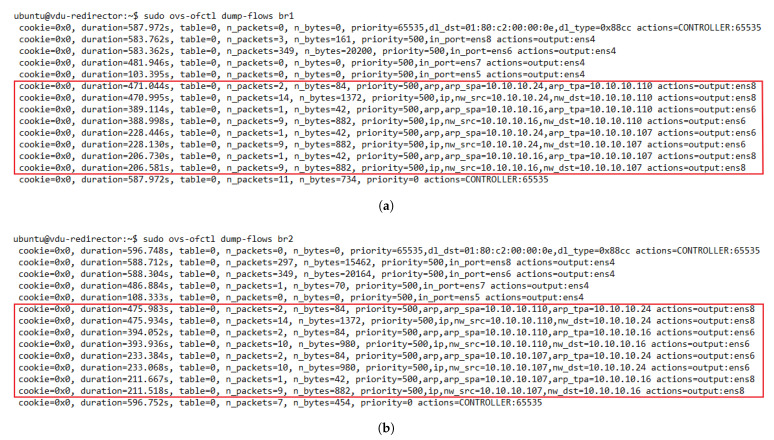
Dynamic flow rule creation in the OFS redirectors with bidirectional flow affinity. (**a**) Flow table of OFS redirector 1. (**b**) Flow table of OFS redirector 2.

One essential requirement of our solution was the assurance of bidirectional flow affinity. To validate the solution for this feature, we generated traffic between the access and internet TGs. We configured the redirector application to create ARP and IP traffic flow rules based on their source and destination addresses. Figure 9 depicts the dynamic configuration of the flow tables in the OFSs. Initially, the switches only had flow rules associated with their interfaces that connected with the transparent VNFs.

Additionally, they also had a default rule configured when they were added to the SDN controller to send packets that did not match any entries in their flow tables to the controller. As new traffic went through the switches, new rules were created according to the established matching criteria. By comparing the flow tables of the switches, we noticed the presence of bidirectional flow affinity, since each pair of source-destination addresses was configured to go through the same port (VNF instance) in both switches. Thus, bidirectional flow affinity was guaranteed by creating the rules in the access and internet side switches simultaneously during the arrival of PACKET_IN events in the SDN controller.

#### 5.2.2. Load Balancing

A load-balancing strategy based on CPU utilization (i.e., least loaded) was selected to evaluate the traffic distribution among members of the transparent cluster. For this test, 20 TCP flows were generated with a bandwidth of 1 Mbps, a duration of 2100 s and a waiting time of 90 s between each flow. For the traffic generation, a script based on iperf3 [47] was implemented to ensure several connections between a pair of TGs located in different subnets. Additionally, a new matching criterion was configured in the SDN controller to redirect traffic based on the five-tuple parameters.

**Figure 10:**
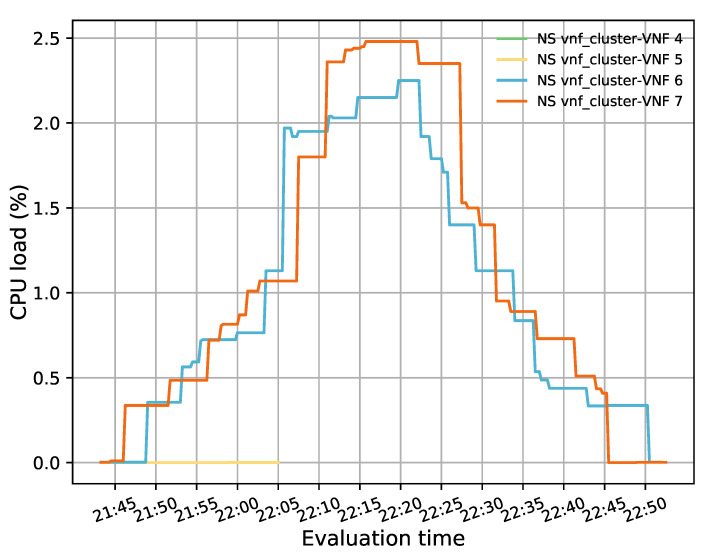
CPU load distribution among members in the transparent VNF cluster.

Figure 10 summarizes the CPU load of each member of the transparent cluster. This figure shows how the CPU utilization of the two active VNF instances, represented in blue and orange colors, increased and decreased in a balanced manner according to the number of active flows passing through them. The blue and orange VNFs had average values of 1.01% and 1.11%, respectively. Thus, the average imbalance achieved by the system was below 10%. This difference was mainly caused by the presence of an odd number of flows in the system and was reduced by the redirector application with the assignment of the next incoming flows to the least loaded VNF instance.

#### 5.2.3. Auto-Scaling Actions

One of the main advantages of virtualization is to provide network operators with the capabilities to increase or decrease their NFs according to specific metrics. This section evaluates our solution by studying its behavior during different scaling actions. Similar to the previous experiments, our pool started with two active members, and the rest were on standby. The TG in the access side generated 50 TCP flows with a bandwidth of 20 Mbps, a connection time of 15,000 s, and a waiting time of 300 s between each flow. These conditions guaranteed that there would be enough flows to increase the CPU load of the active cluster’s members and trigger the scaling actions.

**Figure 11:**
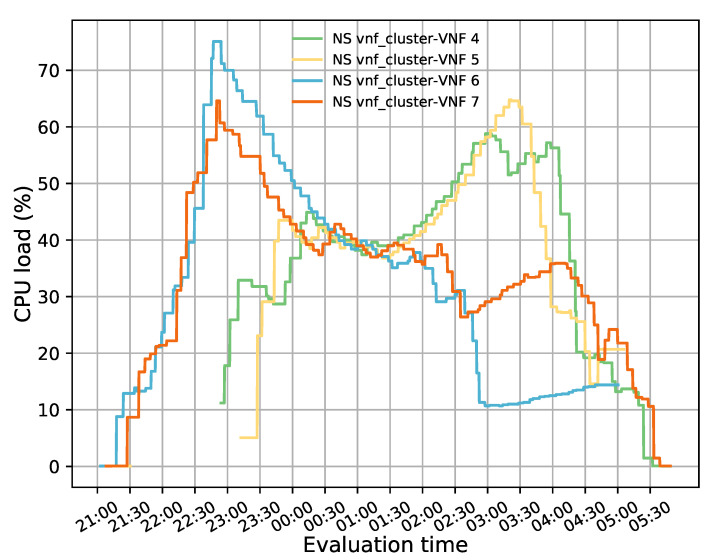
CPU load distribution among members in the transparent VNF cluster while auto-scaling actions were performed.

Figure 11 illustrates the evolution of the test over time. For this experiment, minimum and maximum thresholds of 20% and 50% in the average CPU utilization were established to trigger scale-in and scale-out actions, respectively. Additionally, a threshold time of 120 s and a cooldown time of 300 s were considered. During this test, a total of four scaling actions were executed (i.e., two scale-out and two scale-in). At the beginning of the test, there were only two active instances, represented in blue and orange lines, with very low utilization. However, as time passed, the CPU load increased as new packets were injected into the system. At around 22:40 h, the average CPU load was above 50%, triggering a scale-out action. As a result of this procedure, a new instance was activated (green line), and a second instance (yellow line) was also started some minutes later (after 23:00 h) since the average CPU load of the active VNFs once again passed over the maximum established threshold. Due to the granularity used (i.e., 300 s) in OpenStack, the aggregation metric associated with the new instances took some time to be reflected in Grafana. Hence, the starting value of their CPU usage is different from 0% because they were already processing traffic.

During the second half of the experiment, the oldest flows assigned to the initially deployed VNFs (i.e., blue and orange lines) started to reach their specified lifetime. Thereby, these VNFs were finished by the TG located on the access side. The termination of these flows produced a decrease in the load of these VNFs. Similar effects were noticed in the two last activated instances (i.e., green and yellow lines) around 03:45 h. The average CPU load in the transparent cluster started decreasing until the minimum established threshold was reached. At this point, the instance represented by the blue line was deactivated, and its existing flows were reassigned to the other VNFs due to a scale-in action. After waiting for the completion of the cooldown time, another scale-in action was triggered since the average CPU usage was still below 20%. This action resulted in the deactivation of the transparent VNF depicted in yellow.

**Figure 12:**
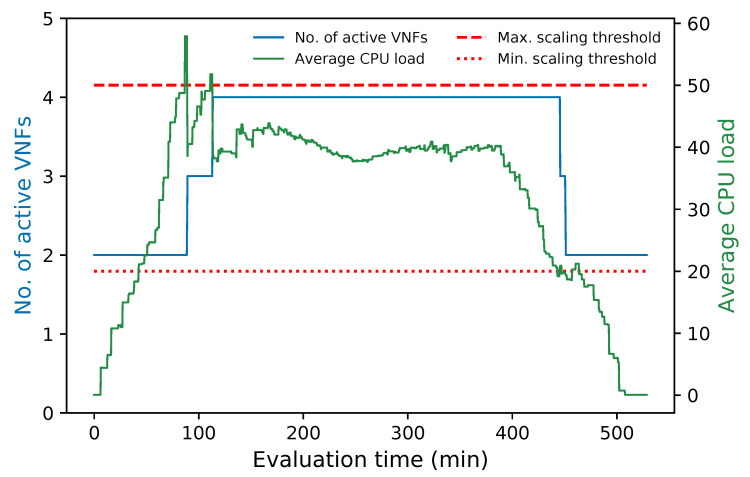
Number of active members in the cluster and average CPU utilization vs. time.

Figure 12 summarizes the aforementioned description. It depicts the variation of the average CPU load in the cluster along with its number of active members during the whole experiment. The figure shows that every time the average CPU was above the maximum threshold (i.e., 50%) a new instance was activated to decrease this parameter. In contrast, at times when the average CPU was below 20%, the number of cluster instances was reduced until the minimum number of active VNFs was reached by the end of the experiment. Additionally, the number of active instances remained constant during the experiment while the average CPU metric was within the established thresholds.

During this experiment, we noticed a reduction in the CPU load of the initially deployed VNFs when new instances were activated even though their allocated flows were invariably maintained. This behavior was due to an increase in system congestion levels caused by the establishment of new connections. To avoid congestive collapse, TCP implements a congestion window (CWND). This mechanism limits the number of outstanding unacknowledged packets that can be in transit on the network for a given pair of source and destination. Specifically, when the network traffic increases, each client that injects TCP packets increases its CWND to reduce its forwarding rate [48]. These reductions are reflected in the CPU load of the BITW VNFs, as the transmission of TCP streams consumes most of the CPU resources [49]. In particular, the newly arrived flows were assigned to the last activated instances as they were the least loaded. Thus, the variation in its CPU load was mainly due to the arrival of new flows and not to the connections already established. In the middle of this test (i.e., 00:30–01:30), the CPU load of the cluster’s members was more balanced, though with slight variations, because the incoming traffic was evenly distributed across all the instances, and had similar utilization levels. However, this behavior did not last too long, and the CPU load of the last activated instance increased while that of the initial VNFs decreased. This reduced the CWND of connections while increasing their send rates, resulting in higher CPU load on scaled instances. The latter was not so noticeable in the first pair of deployed instances due to completing most of its allocated sessions.

#### 5.2.4. Health Monitoring

**Figure 13:**
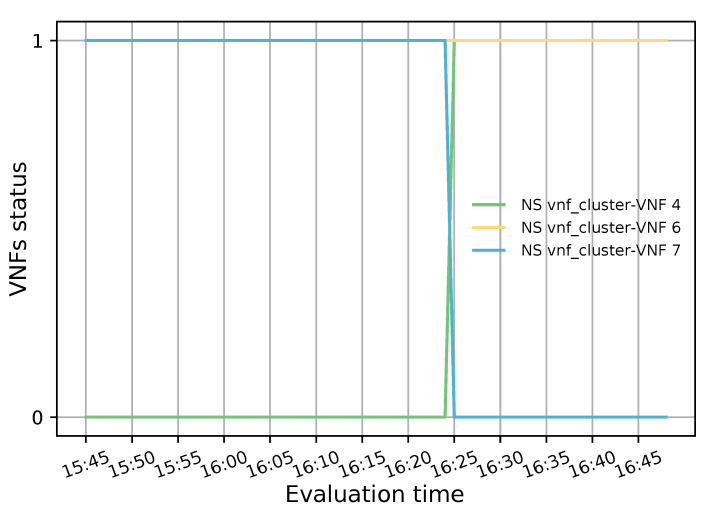
Status of each member belonging to the transparent cluster.

The primary purpose of the health monitoring test was to assess the system’s responsiveness when a member of the transparent VNF cluster fails. To this end, 20 TCP flows were generated with a bandwidth of 20 Mbps, a connection time of 6000 s and a waiting interval of 300 s between each generated flow. Additionally, a low granularity (300 s) was used in OSM and OpenStack to aggregate the collected metrics. This experiment started with two active VNFs to which the incoming TCP traffic was evenly distributed according to their CPU load. After 40 min of the simulation, a failure was emulated in a VNF instance (i.e., VNF with id 7 in the NS) by putting it on standby. This alteration in the selected VNF state can be observed in Figure 13 where the VNF value changed from 1 to 0 just before 16:25 hours (1 means healthy and 0 unhealthy). Additionally, it can be observed that a new instance was activated almost immediately (in less than a minute) to replace the failed VNF and meet the minimum number of active members in the pool.

**Figure 14:**
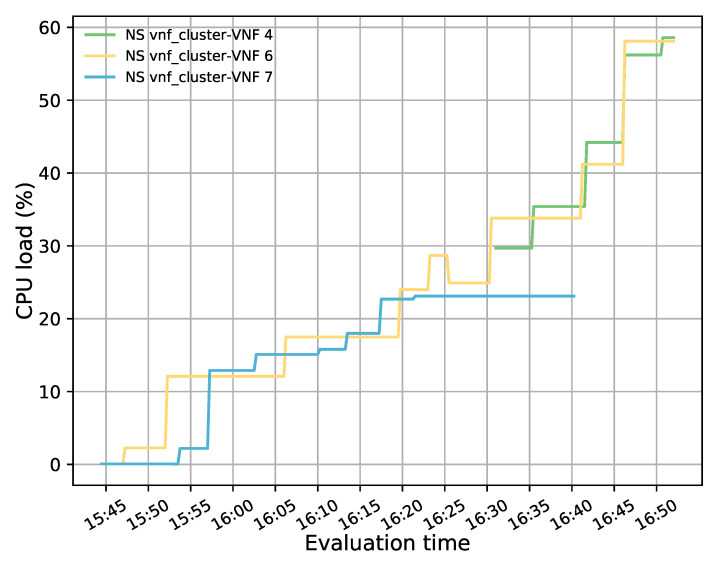
CPU utilization of each active member in the transparent VNF cluster.

**Figure 15:**
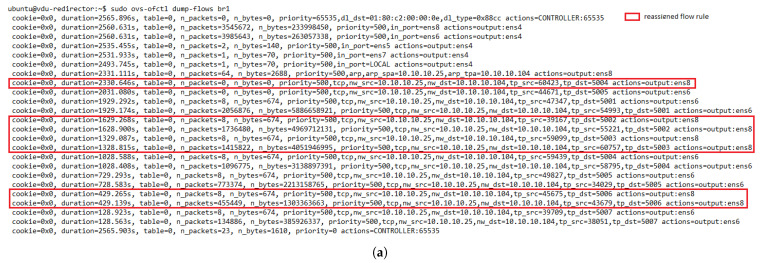

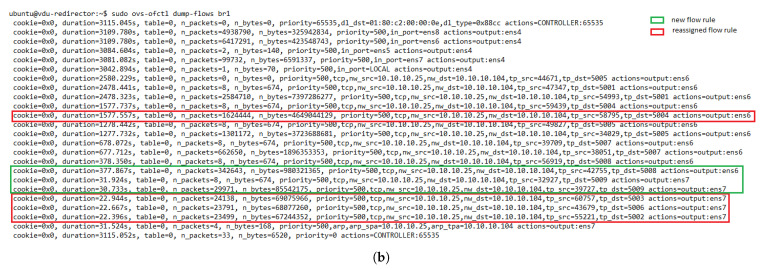
Flow table of redirector 1. (**a**) Before the failure of the VNF instance 7. (**b**) After the failure of the VNF instance 7.

Figure 14 represents the load distribution on each active VNF. Here, the activation of a new VNF instance can be noticed after the failure event. It should also be noted that this new instance had higher CPU utilization than the failed one, as it processed part of the affected traffic along with the newly generated traffic. The latter is evidenced by taking a closer look at the flow rules created in the redirectors (e.g., OFS redirector 1) before and after the failure, see Figure 15a,b, respectively. Comparing both graphs shows that most of the traffic assigned to the failed instance has been redirected to the new VNF (i.e., the flow with the destination port, tp_dst, 5003, 5006, and 5002) along with part of the new incoming traffic (i.e., flow with tp_dst 5009). A small portion of the affected traffic (i.e., flow with tp_dst 5004) was reassigned to the other VNF instance since the new one was not active when the traffic arrived. From Figure 15b, it can also be observed that the flows assigned to the healthy instance (VNF connected in the interface ens6 of the OFSs) maintained their affinity throughout the experiment.

Moreover, some significant delays were noted in displaying the CPU metric in the Grafana dashboard. We believe that better performance can be achieved by using higher granularity values (e.g., every second or minute instead of every five minutes). However, this involves modifying various parameters and configuration files at both OSM and OpenStack levels, which is not trivial and lacks detailed documentation.

## 6. Conclusions

In this paper, we have proposed an SDN-based solution to manage a cluster of transparent VNFs dynamically. Specifically, our solution implements a modular application that runs on an SDN controller to perform load-balancing and auto-scaling decisions. The load-balancing block guarantees bidirectional flow affinity without packet modification by simultaneously configuring OpenFlow rules in the switches comprising the proposed solution. Most of the reviewed literature on this topic conducted experiments by using simulation tools such as Mininet. In contrast, our solution was implemented in a real environment using two well-known frameworks, OSM and OpenStack.

The evaluation results validated the feasibility of the proposed solution. The solution was shown to successfully redirect E2E traffic through the transparent cluster without losing any packets. Additionally, its bidirectional flow affinity capability was demonstrated by comparing the switches tables and finding each pair of source-destination addresses attached to the same port. Likewise, the effectiveness of the load-balancing and auto-scaling mechanisms was demonstrated through different experiments. Moreover, the monitoring module’s performance was evaluated through a health test in which we could notice the activation of a new instance to meet the minimum number of active members in the cluster after detecting a failed member.

Future work directions for this topic include designing strategies based on traffic forecasting. These strategies could enable scaling and load-balancing decisions to adapt to dynamic variations in traffic proactively. Furthermore, we intend to implement and test the performance of the proposed solutions in other VIM (e.g., Kubernetes) and MANO technologies to evaluate and compare their performance and extend the evaluation scenario (network service topology and SFC).

## Figures and Tables

**Table 1:** Hardware and software specifications.

	Description	Specifications
Server 1	NFVI with OpenStack Controller	Memory: 32 GB RAM DDR4
		Processor: Intel Core i7-5820K CPU @ 3.30 GHz
		OS: Ubuntu Server 18.04.2
Server 2	NFVI with OpenStack Compute	Memory: 16 GB RAM DDR4
		Processor: Intel Core i7-5820K CPU @ 3.30 GHz
		OS: Ubuntu Server 18.04.2
OSM host	HP Compaq 8100 Elite SFF PC	Memory: 8 GB RAM DDR3
		Processor: Intel Core i5 CPU 650 @ 3.20 GHz
		OS: Ubuntu Desktop 18.04.2
OpenStack		Train
OSM		Release EIGHT

## Data Availability

Not applicable.
